# Mixing Linear Polymers with Rings and Catenanes: Bulk and Interfacial Behavior

**DOI:** 10.1021/acs.macromol.3c01267

**Published:** 2023-10-03

**Authors:** Roman Staňo, Christos N. Likos, Sergei A. Egorov

**Affiliations:** † Faculty of Physics, University of Vienna, Boltzmanngasse 5, 1090 Vienna, Austria; ‡ Vienna Doctoral School in Physics, University of Vienna, Boltzmanngasse 5, 1090 Vienna, Austria; ¶ Department of Chemistry, University of Virginia, Charlottesville, Virginia 22901, United States; § Erwin Schrödinger International Institute for Mathematics and Physics, Boltzmanngasse 9, 1090 Vienna, Austria

## Abstract

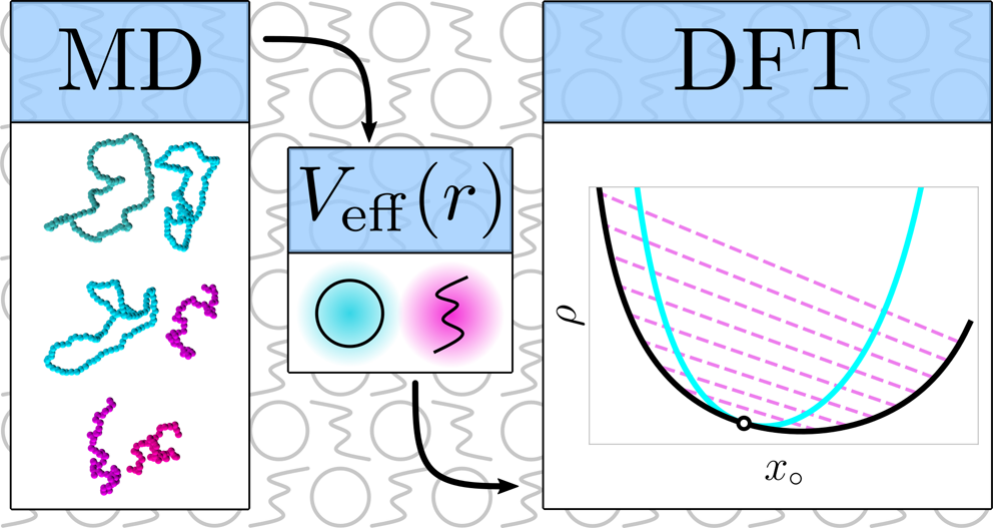

We derive and parameterize effective interaction potentials between a multitude of different types of ring polymers and linear chains, varying the bending rigidity and solvent quality for the former species. We further develop and apply a density functional treatment for mixtures of both disconnected (chain–ring) and connected (chain-polycatenane) mixtures of the same, drawing coexistence binodals and exploring the ensuing response functions as well as the interface and wetting behavior of the mixtures. We show that worsening of the solvent quality for the rings brings about a stronger propensity for macroscopic phase separation in the linear-polycatenane mixtures, which is predominantly of the demixing type between phases of similar overall particle density. We formulate a simple criterion based on the effective interactions, allowing us to determine whether any specific linear-ring mixture will undergo a demixing phase separation.

## 1 Introduction

Recently, there has been a growing interest in research of polymers with complex architectures,^1,2^ such as ring polymers. Binding the two ends of a linear chain together, thereby turning the chain into a ring, creates a permanent topological invariant, since bonds of such rings can never cross each other,^3,4^ which comes with associated entropic penalty. The above constraints dramatically affect the resultant macromolecular properties, for example, the radius of gyration, *R*_g_. Conformations of a single, ideal linear chain follow random-walk statistics, resulting into scaling relation *R*_g_ ∼ *N*^0.5^, where *N* is the polymerization degree.^5^ On the other hand, a single ring exhibits *R*_g_ ∼ *N*^0.588^ with the Flory critical exponent,^6^ even for a ring with no excluded volume interactions. Moreover, in the concentrated melts, the chains anew obey the random-walk statistics, while rings^7−11^ adopt non-Gaussian conformations of collapsed globules, reminiscent of randomly branched trees,^12^ which can be threading each other,^13−15^ albeit the role of threading entanglement has not been fully unraveled yet. Arguably, the most prominent consequence of ring topology manifests itself in the dynamics^16^ and viscoelastic response^17−21^ of the polymer melts. For the former, long linear chains relax predominantly via one-dimensional diffusion by reptation^22^ in a tube along the contour of the chain, while rings possessing no ends utilize different modes of relaxation.^23^ For the latter, and for the case of low-molecular-weight rings, the stress relaxation modulus of ring polymers exhibits a power law in a long range of frequencies, with no signs of rubbery plateau, present for linear chains.^24,25^ On the other hand, it has been recently found that for high-molecular-weight rings, an unexpected rubbery plateau reappears, which has been tentatively attributed to strong ring–ring interpenetration and caging.^26^ It is important to realize that the above differences are brought about purely by differences in the global topology of a macromolecule, while the chemistry of the monomeric units is the same. Fundamental understanding of the above effects is important, mainly because ring polymers are prevalent in nature in the form of plasmids or bacterial DNA, and furthermore, topological constraints play an important role in organization of linear chains as DNA in human genome^27−29^ as well.

Topological constraints go beyond having just a chain or a ring.^30^ Notably, the ring topology allows for permanent linking of two such macromolecules via a Hopf link,^31^ forming [2]catenanes,^32−35^ with prospective application as components of molecular machines^36,37^ or in medicine.^38^ Catenane links show higher elasticity than their covalently bonded counterparts,^39^ predestining them for usage in elastomer materials, exemplified by olympic gels^40^ or slide-ring gels^41,42^ or even in biological structures such as kinetoplast DNA.^43−48^ The latter are sheets of thousands of catenated DNA rings, resembling chain-mail armor, naturally occurring in unicellular organisms of the class Kinetoplastida. Recent experiments suggest that when digesting the kinetoplasts using enzymes cutting the DNA rings, one first breaks the percolation in the interior of the sheet and is left with the periphery, now being a ring of rings.^49,50^ Better understanding of such poly[*n*]catenanes,^51,52^ either cyclic or open, presents one of the main challenges for the topological soft materials. Simulations predict that the structure of poly[*n*]catenanes in melts is similar to linear chains in the limit of long time scales and length scales but shows a different behavior at the shorter scales,^53,54^ with a similar relaxation time decoupling being present also in dilute solutions.^55−57^ However, experimental verifications for some of the simulation predictions are at the moment missing, mainly due to the lack of high-yield synthetic methods leading to high-molar-mass poly[*n*]catenanes.^58^

Similarly to poly[*n*]catenanes, synthesis of ring polymers is accompanied by challenges,^59^ most notably possible contamination of samples of rings by linear chains. It is worth mentioning in this context that metastable polymer chemistry rules out the existence of linear chains in a ring solution or melt, as cleavage of a ring rapidly leads to a degradation to its monomers.^26,59^ It has been shown that even an addition of ∼1 wt % of linear counterparts can alter the dynamics of ring melt, reintroducing the elastic plateau in the stress relaxation functions.^24^ In such blends, chains can thread through the rings, restricting their lateral modes, decreasing their diffusion coefficient, while significantly increasing the zero-shear viscosity.^60^ To understand the interplay of ring and chains, a great variety of model systems of blends have been devised^61−67^ and explored from the standpoint of rheology. However, research in the direction of thermodynamics and structure is mostly missing. Under which conditions are the ring and chains miscible, hence forming stable equilibrium blends? Recently, it has been shown, using molecular dynamics and relating structure factor to χ-parameter, that linear/ring melts of chemically similar polymers should be more miscible than ring/ring or linear/linear counterparts.^68^ This has been explained in terms of topological solubilization, arguing that chain–ring threading can increase the conformational entropy of rings. Other theoretical approaches to assessing thermodynamics stability present extensions of Flory–Huggins theory,^69^ including additional contributions for topological volume,^70,71^ all of which focus mainly on large *N* limit. In our study, however, inspired by the catenane systems, we focus mainly on shorter rings, which should have a limited window of miscibility with chains, even if the two have chemical dissimilarity. Specifically, we vary two parameters of the rings, quality of the solvent^72,73^ and backbone stiffness,^56^ both of which are known to affect the phase behavior of pure linear chains.^5,74^

In the current work, we present an approach, different to the ones listed above, based on classical density functional theory (DFT), similar to our previous works.^75,76^ We describe blends of linear polymers with ring polymer and catenanes as mixtures of two types of blobs representing a subsection of the chain and an individual ring, respectively. First, we use monomer-resolved molecular dynamics to derive effective potentials for all combinations of blob types, hence turning the phase of polymers into a binary liquid of soft particles. Within the mean-field approximation, we use DFT to calculate phase diagrams, structure of bulk phases, and interfaces between them, all of the above for a set of different polymer flexibilities and solvent quality, while simultaneously assessing validity of used approximations.

## 2 Results and Discussion

### 2.1 Microscopic Models and Coarse-Graining

As the complexity of ring-linear and catenane-linear mixtures is enormous, our purpose is to coarse-grain the mixtures by modeling both linear chains and rings as blobs represented by their centers of mass. Accordingly, in simple ring-linear mixtures, each linear chain and each ring will be represented by a single effective coordinate each: their corresponding center of mass. We index chain blobs as (1) and ring blobs as (2), the former blobs consisting of *N*_1_ = 50 monomers and the latter ones of *N*_2_ = 100 monomers, resulting in blobs of similar spatial extent. For mixtures of long chains and catenanes of similar size, on the other hand, each long molecule will be represented by an array of *M*_*i*_ connected blobs, *i* ∈ {1, 2}, in the spirit of the previously employed multiblob representations of polymers,^77−81^ extended now to polycatenanes. We will consider only moderate values of *M*_*i*_ in what follows, whereby *M*_1_ = *M*_2_ = 1 corresponds to linear-ring mixtures and *M*_2_ > 1 corresponds to linear-catenane blends.

The quantities of central importance are the pairwise effective potentials, *V*_11_(*r*), *V*_12_(*r*), and *V*_22_(*r*), between the various blobs, entering the theoretical analysis employed below. For the underlying monomer-resolved model, we consider a linear chain of length *N*_1_ = 50 monomers and an unknotted ring of length *N*_2_ = 100 monomers, both being modeled by means of a standard bead–spring coarse-grained polymer model.^82^ All monomers are represented by point particles interacting with the nonbonded potential *U*_*ij*_(*s*), similar to the one introduced by Weeks, Chandler, and Andersen (WCA)^83,84^
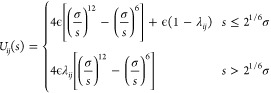
1where *s* is the distance between the two monomers, *i*, *j* ∈ {1, 2} and we set the length scale σ = 1 and energy scale ϵ = *k*_B_*T* = 1/β = 1, respectively. The parameter λ_*ij*_ controls the depth of the attractive well of the potentials. For the interactions between monomers in the chain and also those between chain and ring monomers, they are set to λ_11_ = λ_12_ = 0, thereby corresponding to the purely repulsive WCA interaction and modeling athermal solvent conditions. In general, however, the chemistry of the ring and chain monomers can be different. To explore the effect of worsening solvent quality for the ring monomers on the effective ring–ring and ring–chain interactions, we allow for nonvanishing attractive tails in the ring-intermonomer potential *U*_22_(*s*), using λ_22_ ∈ {0.0, 0.1, 0.2, 0.3}, which nevertheless still correspond to good solvent conditions.^85^ Finally, we note that we use a truncated and shifted version of the potential of eq 1, with a cutoff of 2.5σ.

Connectivity of successive monomers in the polymer is governed by finitely extensible nonlinear elastic (FENE) potential
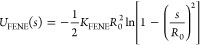
2where *K*_FENE_ = 30*k*_B_*T*/σ^2^ and *R*_0_ = 1.5σ. The combined potential well of the above two potentials gives rise to uncrossable bonds of mean lengths of ⟨*l*⟩ ≈ 0.96σ. Finally, the chains are fully flexible (κ_1_ = 0), whereas for rings we employ a harmonic cosine potential *U*_bend_(ϕ) acting on the angle ϕ between two successive bonds, emulating bending stiffness:
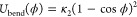
3Here, β*κ*_2_ ∈ {0, 5, 10} is the bending spring constant and ϕ is the instantaneous angle between consecutive bond vectors. The above bending constant translates to persistence lengths *l*_per_/⟨l⟩ ∈ {0, 3.4, 4.9} or Flory ratios *C*_∞_ ∈ {1.0, 6.6, 9.4}, respectively, as detailed in ref (86)

Ultimately, our model has two free parameters which we change: the stiffness of the ring and the strength of the attractive interaction of ring–ring monomers. We select four specific points in this two-dimensional parameter space, as listed in Table 1. The choice of this particular set of parameters is deliberate since they lead to macrophase separation in the realm of our theoretical approach, indicated by the fact that the product *ab* > 1 in eq 18, as we will expound later. We also tried many different parameter combinations, summarized in Table S1 in the Supporting Information (SI).

**Table 1:** Table Listing the Parameters of the Studied Systems^a^

	*U*_bend_(ϕ)		*U*_*ij*_(*s*)			*V*_11_(*r*)		*V*_12_(*r*)		*V*_22_(*r*)		
case	βκ_1_	βκ_2_	λ_11_	λ_12_	λ_22_	βε_11_	*R*_11_/σ	βε_12_	*R*_12_/σ	βε_22_	*R*_22_/σ	*ab*
*A*	0	0	0.0	0.0	0.1	3.14	5.55	3.89	6.73	5.43	6.69	1.321
*B*	0	0	0.0	0.0	0.2	3.14	5.55	4.29	6.34	4.86	6.25	1.536
*C*	0	5	0.0	0.0	0.0	3.14	5.55	2.07	9.07	3.27	9.74	1.212
*D*	0	10	0.0	0.0	0.3	3.14	5.55	1.79	9.40	1.07	11.03	2.340

a Stiffness of chain (1), βκ_1_, and ring (2), βκ_2_, depth of the interaction well between monomeric *U_ij_* for chain–chain, λ_11_; chain–ring, λ_12_; and ring–ring, λ_22_, followed by the fitted parameters of the resultant effective potential (see eq 6 and Figure 2) and mixing criterion *ab* (see eq 18).

**Figure 1:**
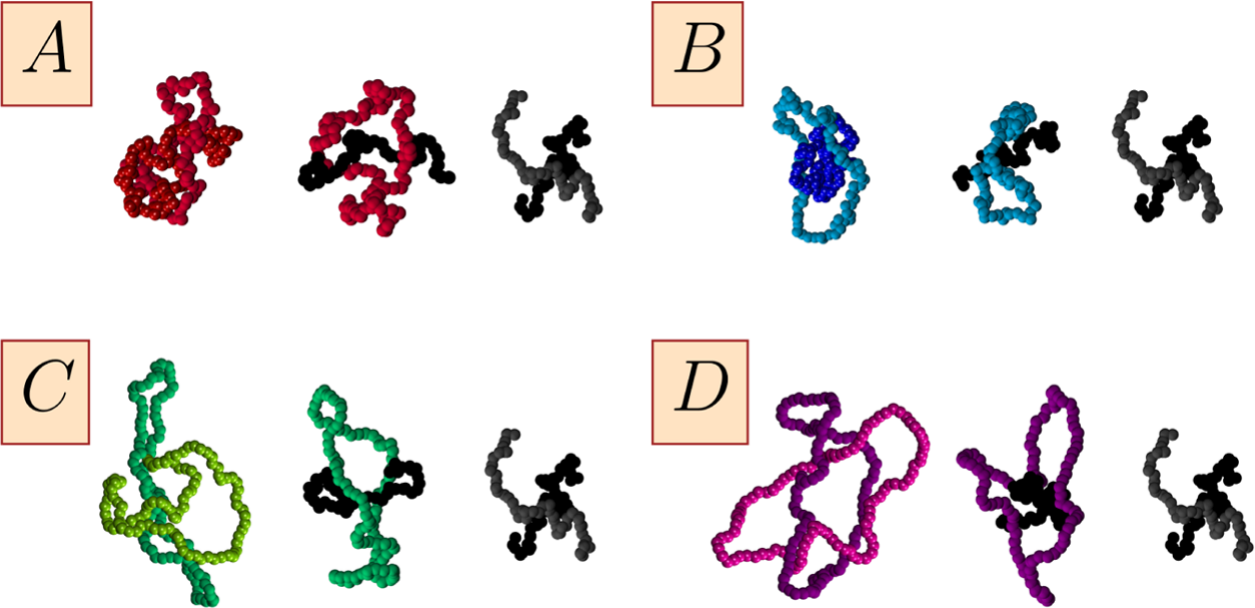
Representative snapshots of monomer-resolved systems at approximately vanishing center-of-mass separation, the coding (*A–D*) corresponding to the four selected cases from Table 1. For each of the four triplets, the left panel shows typical conformations of two rings, which are rendered in slightly different shades of color and glossiness for better visibility. The middle panels show conformations between chain (black) and ring and the right panel conformations of two chains.

To determine the effective potentials *V*_*ij*_(*r*) between the centers of mass of the blobs for each of the four cases *A*, *B*, *C*, and *D*, we independently constructed the three following systems: one with two chains, one with two rings, and finally a system with one chain and one ring as depicted in Figure 1. Each system is placed in a cubic simulation cell of size *L* = 150σ, assuring that a macromolecule does not interact with itself through the periodic boundary conditions. For each of the systems mentioned above, we applied a biasing (umbrella) potential
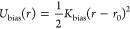
4between the centers of mass of the two macromolecules, where *K*_bias_ = 2.5*k*_B_*T*/σ^2^, and we carried out independent simulation for each of *r*_0_/σ ∈ {0.0, 0.5, 1.0, ···, 30.0}; characteristic snapshots of the interacting polymers for *r*_0_ = 0 are shown in Figure 1. The system was evolved using the LAMMPS^87^ implementation of Langevin dynamics with friction coefficient γ = 1/τ and time step δ*t* = 0.01τ, where τ = (σ^2^*M*_o_/*k*_B_*T*)^1/2^ = 1 is the unit of time, where *M*_o_ = 1 is mass of monomer. A typical run was 5 × 10^6^τ long, and the simulations were analyzed using the weighed histogram analysis method (WHAM),^88,89^ removing the bias and yielding the pair correlation function *g*_*ij*_(*r*) between the two macromolecules in the high dilution limit, which can be related to the effective potential as
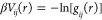
5For more details on the procedure and our workflow, we refer the reader to our previous publications.^86,90−93^ Finally, the effective potentials obtained from the monomer-resolved simulations were fitted using the generalized exponential model (GEM)
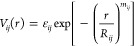
6where ε_*ij*_ and *R*_*ij*_ are fittable parameters setting the energy scale and length scale, respectively, and *m*_11_ = 2, *m*_12_ = 3, and *m*_22_ = 4 as in ref (94). The resulting values of the parameters ε_*ij*_ and *R*_*ij*_ are summarized in Table 1.

**Figure 2:**
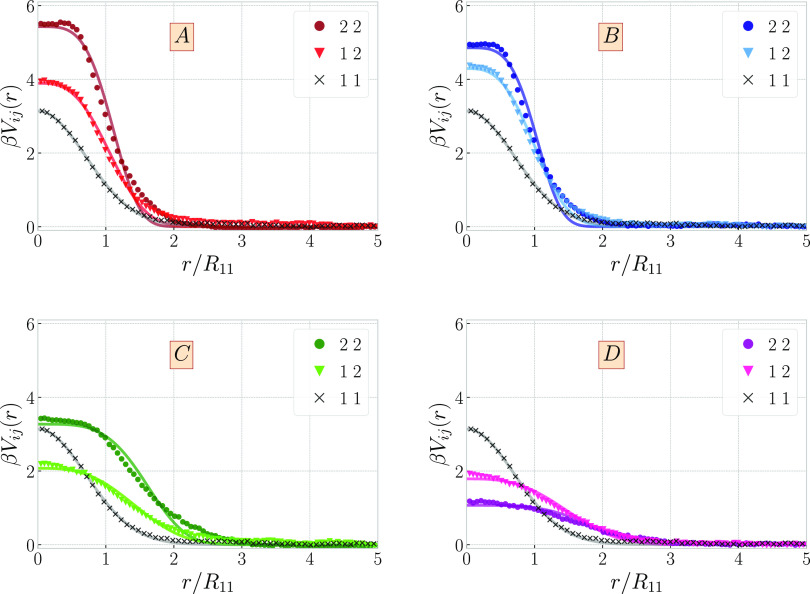
Effective isotropic potentials between two rings (22), two chains (11), and ring and chain (12) as a function of separation between the two molecules. The labels (*A–D*) on the panels correspond to the four selected cases from Table 1. Points were obtained by monomer-resolved simulations, and lines are fit using eq 6. Distance is normalized by the characteristic length scale of the chain–chain interaction, *R*_11_. Analogous figures for additional combinations of parameters can be found in Figure S1 in the SI.

Overall, the derived potentials displayed in Figure 2 show satisfactory agreement between the monomer-resolved potentials and the GEM forms for all four cases. The effective interaction between the two chains is effectively a Gaussian centered at zero separation,^95,96^ while rings exhibit a pronounced plateau at *r* → 0 as established earlier.^97−99^ The two effective interactions *V*_12_(*r*) and *V*_22_(*r*) offer us valuable insights into the ways in which the physical (λ_22_) and chemical (βκ_2_) characteristics of the rings affect the outcomes. Comparing models (A) and (B) in Figure 2(A),2(B), we see that worsening the solvent quality for the ring beads from λ_22_ = 0.1 to λ_22_ = 0.2 has two antagonistic effects. On the one hand, the inter-ring potential *V*_22_(*r*) becomes weaker, as the enhanced propensity of the monomers to attract counteracts the steric entropy loss at close separations. On the other hand, the ring-linear potential *V*_12_(*r*) becomes stronger since now the internal monomer density of the shrunken ring grows and with it the entropic penalty of placing the centers of mass of the ring and the chain on top of each other. More quantitatively, we find β*V*_22_(0) ≅ 5.4 for λ_22_ = 0.1 and β*V*_22_(0) ≅ 4.9 for λ_22_ = 0.2, in comparison to β*V*_22_(0) ≅ 6.0, observed for the rings with λ_22_ = 0 in ref (99). Turning our attention to the effect of stiffness, which grows from βκ_2_ = 5 for the system *C*, Figure 2(C), to βκ_2_ = 10 for the system *D*, Figure 2(D), we see that in this case both *V*_22_(*r*) and *V*_12_(*r*) become weaker, as the increased bending rigidity opens the rings up and creates more space for the penetration of other rings or linear chains, thereby reducing the associated entropic overlap penalty. In Figure S1 of the SI, we show the effective potentials obtained for a variety of other parameter combinations, shown in Table S1.

### 2.2 Density Functional Theory

Our theoretical investigations of the bulk, structural, and interfacial properties of linear-ring and linear-catenane mixtures are based on density functional theory (DFT). The key quantity in this formalism is the grand potential^100^ Ω[ρ_1_, ρ_2_], which in the present case is a functional of the (generally inhomogeneous) densities of the two components, ρ_1_(**r**) and ρ_2_(**r**). Here, a component has the meaning of a coarse-grained blob describing either a linear chain or a ring, i.e., precisely the quantities for which we derived the effective potentials *V*_*ij*_(*r*) in the preceding section. The grand potential contains contributions from the ideal free energy, which quantify the entropy loss for any deviation of the densities from the homogeneous profile, the excess free energy, which contains all energy and entropy contributions due to interblob interactions, the influence of external potentials *W*_*i*_(**r**) such as walls or gravity, and the chemical potentials μ_*i*_, *i* ∈ {1, 2} of the two species. For the system at hand, we employ the grand potential given by the expression
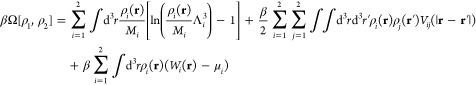
7where Λ_*i*_ is the de Broglie wavelength of species *i*. The grand potential of eq 7 involves certain approximations, which we discuss below.

Whereas the ideal free-energy contribution (first term on the right-hand side of eq 7) is exact, the functional of eq 7 features an excess free energy (second term on the right-hand side) which is bilinear in the density fields with an integration kernel given solely by the effective interactions *V*_*ij*_(**r** – **r**′|). This mean-field/random-phase approximation for the excess free energy has been demonstrated to be very accurate for soft interactions that do not diverge at the origin both for one-component systems^101−103^ and for binary mixtures.^94,104,105^ The far-reaching theoretical predictions of this form regarding the self-assembly of such ultrasoft-particle systems^103,106^ have been recently confirmed experimentally.^107^

In writing down the excess free energy in eq 7 and applying it at all polymer densities in what follows, we made the assumption that the pairwise effective interactions remain valid in the domains in which the relevant phenomena (such as phase separation) take place. Although this is an approximation, it is not a drastic one. Indeed, the validity of the ring–ring effective potentials was tested in our previous studies, where we compared the resulting pair correlation functions of the effective model with fully monomer-resolved simulations in solutions in a broad range of densities. We found out that for flexible rings,^97^ the effective potentials are quite accurate up to concentrations of ρ/ρ* ≲ 4, where ρ* is the overlap concentration, and for stiff rings^90^ up to ρ/ρ* ≲ 3, which can be pushed even to ρ/ρ* ≲ 5, when using anisotropic effective potentials.^91^ Similarly, for the chain–chain case,^96^ the effective potentials are accurate up to several overlap concentrations. While we have not tested the accuracy of the mixed ring–chain potentials, we expect them to have the same range of accuracy as those of the above pure solutions. Taking the condition ρ*R_11_^3^ = 1 as an estimate for the polymer overlap density ρ* in the solution, it can be see that the critical points as well as large parts of the binodals and spinodals in Figure 4 reside below ρ*, where the effective potentials are indeed accurate. This condition is fulfilled even more strongly for the phase diagrams of the chain-catenane micelles shown in Figure 5. As regards ways to accurately deal with even higher densities, the appropriate technique is to follow a multiblob coarse-graining, along the lines of, e.g., refs (77,80)

An additional approximation involved in the grand potential form of eq 7 concerns the treatment of the connectivity of linear chains consisting of *M*_1_ linear blobs and/or catenanes consisting of *M*_2_ ring blobs. Whereas this feature is taken into account in the ideal part of the Helmholtz free energy in which the quantities ρ_*i*_(**r**)/*M*_*i*_ appear, it is absent in the excess term, in which only interactions of blobs with neighboring ones show up, independent of whether these blobs are connected or not. To test the accuracy of this approximation, we compare here its predictions for the binary mixture equation of state with the corresponding results of the Polymer Reference Interaction Site Model (PRISM) integral equation theory,^84,108^ where the Ornstein–Zernike (OZ) equation is solved in conjunction with an (approximate) closure relation, and the chain connectivity is explicitly taken into account via the intramolecular pair correlation functions *w̃*_*ii*_(*k*) in reciprocal space, as defined below.

In PRISM theory, each polymer in a concentrated solution is considered as a sequence of interacting sites in a spirit similar to that of interacting particles (monomers) for the case of simple liquids. For binary polymer blends, one introduces the radial distribution functions *g*_*ij*_(*r*) and the total correlation functions *h*_*ij*_(*r*) = *g*_*ij*_(*r*) – 1, *i*, *j* ∈ {1, 2}. Chain connectivity is taken into account by the intramolecular pair correlation functions *w*_*ii*_(*r*), *i* ∈ {1, 2} (for homopolymers, the *w*_*ij*_(*r*) functions with *i* ≠ *j* vanish). The OZ equation takes in Fourier space a matrix form that reads as^84,108^
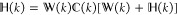
8where  and  are the matrices that are constructed from the Fourier transforms of the total correlation functions *h*_*ij*_(*r*) and the direct correlation functions *c*_*ij*_(*r*), respectively. The diagonal matrix  contains the Fourier transforms of the intramolecular pair correlation functions *w*_*ii*_(*r*), for which we use the freely jointed chain model *ŵ*_*ii*_(k) = sin(*R*_*ii*_*k*)/(*R*_*ii*_*k*).

In order to solve the system of OZ equations given in the matrix form by eq 8, one needs to supplement them with the corresponding closure relations^84^ given by
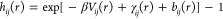
9where γ_*ij*_(*r*) = *h*_*ij*_(*r*) – *c*_*ij*_(*r*) is the indirect correlation function and *b*_*ij*_(*r*) is the bridge function. The exact form of the latter is unknown, and various approximations are available in the literature.^84^ In the present work, we employ the hypernetted chain (HNC) closure given by *b*_*ij*_(*r*) = 0. The PRISM integral equations combined with the HNC closure are solved using the standard Picard iteration procedure.^109^ The real-space grid is discretized with a spacing of Δ*r* = 0.01σ, and 8192 points are used in performing the Fourier transforms. The tolerance parameter for the convergence criterion of the Picard iteration is set to 10^–7^. Having calculated the radial pair distribution functions *g*_*ij*_(*r*) from PRISM-HNC, the pressure is obtained by the expression
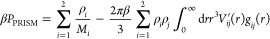
10the prime denoting the derivative with respect to the argument. The MFA-DFT expression for the pressure is given later in eq 15.

**Figure 3:**
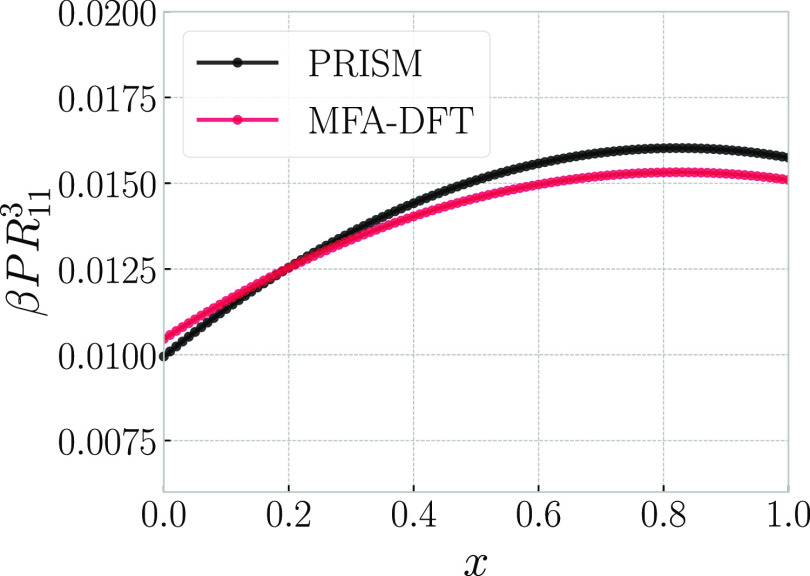
Total pressure of polycatenane (*M*_2_ = 5) and long-chain (*M*_1_ = 5) mixture as a function of ring fraction at total density ρ*R*_11_^3^ = 0.025 plotted for the system *D* from Table 1.

The corresponding PRISM and MFA-DFT results for the dimensionless pressure βPR_11_^3^ are presented in Figure 3 as a function of the ring mole fraction *x* at the overall fixed density ρ*R*_11_^3^ = 0.025 (with *M*_1_ = *M*_2_ = 5 for the system D), the overall agreement is very good and therefore it justifies the approximate form of DFT of eq 7. A major advantage the latter has over PRISM is that it is a general approach valid for both homogeneous and inhomogeneous mixtures, and thus, it allows for the determination of thermodynamic, structural, and interfacial properties all within the same, self-consistent theoretical framework.

### 2.3 Phase Behavior

The inhomogeneous DFT methodology discussed in the previous sections can be readily adapted to study the bulk phase behavior of ring–chain binary mixtures. To this end, one simply replaces the inhomogeneous densities in eq 7 for the grand potential by the corresponding bulk values ρ_*i*_, *i* ∈ {1, 2}. Carrying out the trivial spatial integrals on the first and second terms on the right-hand side of eq 7, we readily obtain the ideal (*f*_id_) and excess (*f*_exc_) Helmholtz free energies per unit volume  as
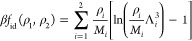
11and
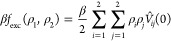
12where *V̂*_*ij*_(0) is the zero-wavevector Fourier component of the interaction pair potential *V*_*ij*_(*r*), given by^94^
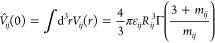
13and Γ(*x*) denotes the γ function. From the above, one obtains the chemical potentials for the two components as follows
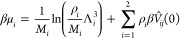
14whereas the pressure of the system is given by
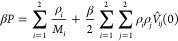
15

Possible phase transitions within the mixture are most efficiently identified by performing a Legendre transform from the Helmholtz free energy, whose natural variables are temperature, volume, and the number of particles *N*_1_, *N*_2_ of the two species, to the Gibbs free energy per particle *g*(*x*, *P*, *T*) which has the mixture composition *x* = *N*_2_/*N*, (*N* = *N*_1_ + *N*_2_), pressure, and temperature as its natural variables. Considering the curves *g*(*x*, *P*, *T*) vs *x* ∈ [0, 1] for fixed *T* along isobars, phase separation is signaled by the existence of nonconvex parts on the same. Convexity is restored by performing the common tangent construction, resulting in a straight-line envelope connecting phases with compositions *x*^I^ and *x*^II^ at the end points of the common tangent. It is straightforward to show that the two resulting phases have common chemical potentials μ_*i*_^I^ = μ_*i*_^II^, *i* ∈ {1, 2}, for each component of the mixture, and since they exist on an isobar and for a common temperature, they satisfy all requirements for macroscopic phase coexistence, i.e., they lie on the binodal.

The occurrence of phase coexistence is equivalent to the appearance of the so-called spinodal line, on which *g*_*xx*_(*x*, *P*, *T*) = 0, each subscript denoting a derivative. The Gibbs free energy per particle cannot be cast in a closed form but we can work instead with the Helmholtz free energy per particle, *f̃*(*x*, *v*, *T*), expressed as a function of the composition *x*, the specific volume , and the temperature *T*, which is easily obtainable in the closed form from eqs 11 and12 as *f̃* = *v*(*f*_id_ + *f*_exc_), also expressing ρ_1_ = (1 – *x*)/*v* and ρ_2_ = *x*/*v*. In this representation, the spinodal line of the binary mixture takes the form^110,111^
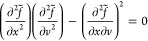
16Using the expressions in eqs 11 and 12, we obtain the spinodal line as
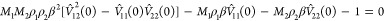
17Introducing two dimensionless variables ζ = *M*_1_ρ_1_β*V̂*_11_(0) and η = *M*_2_ρ_2_β*V̂*_22_(0) and two dimensionless ratios
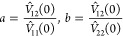
18the equation for the spinodal takes the following simple form
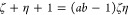
19Since the quantities ζ and η are non-negative, it is evident that eq 19 can only be satisfied if *ab* > 1, justifying our choice of model systems A, B, C, and D to study macrophase separation.

For given values of *a* and *b*, the above equations define a universal curve for all values of the two components chain lengths *M*_1_ and *M*_2_. However, the location of the critical point (ζ_c_, η_c_) for a binary mixture with a given ratio of chain lengths *m* = *M*_2_/*M*_1_ does depend on *m* since
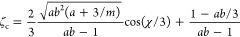
20where
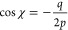
21with
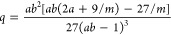
22
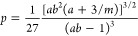
23The second dimensionless coordinate of the critical point is given by
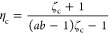
24The above equations show that for given interaction parameters *a* and *b*, the spinodal is a line on the (ζ, η) plane that does not depend on *m*. The location of the critical point on this spinodal, on the other hand, is affected by modifications of the ratio *m* = *M*_2_/*M*_1_ as shown in Figure S2 in the SI.

#### 2.3.1 Linear-Ring Mixtures

**Figure 4:**
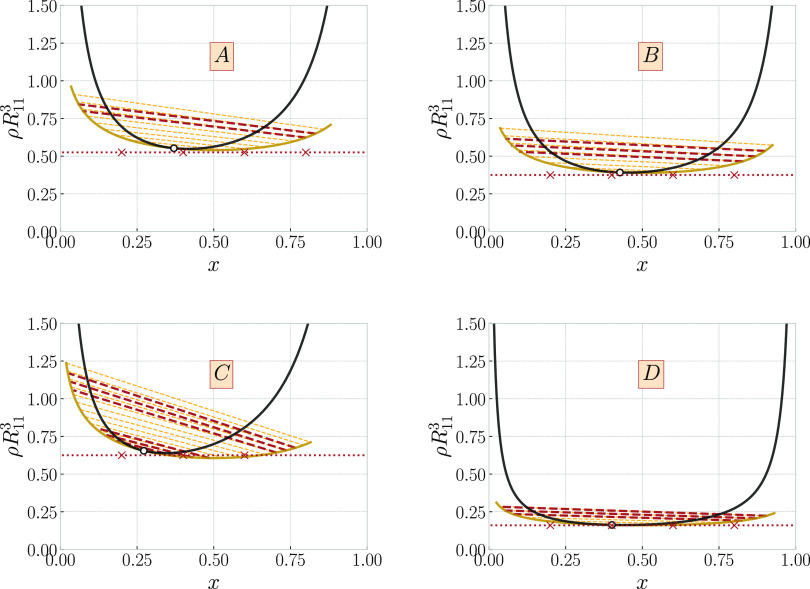
Phase diagrams for mixtures of linear chains (*M*_1_ = 1) and rings (*M*_2_ = 1), plotted on the total density-ring fraction plane, (ρ, *x*). The labels (*A–D*) on the panels correspond to the four selected cases from Table 1. Solid golden lines represent binodals, and solid black lines represent spinodals with the critical point highlighted by a black point with white interior. Dashed golden lines are selected tielines, connecting coexisting points on the binodals. Selected points marked by dark red cross are further explored in Figure 6 and selected tielines highlighted in dark red are further explored in Figure 8.

We start by constructing bulk phase diagrams of binary mixtures A, B, C, and D (with *M*_1_ = *M*_2_ = 1). As discussed above, binodals are obtained numerically by equating the pressures (given by eq 15) and chemical potentials of both components (given by eq 14) in the two phases. Spinodals are given analytically in eq 17. Binodal and spinodal for a given system meet at the critical point, for which analytical result was also given above. The resulting phase diagrams with binodals, spinodals, and tielines connecting coexisting points are plotted in Figure 4 in the variables total (dimensionless) density ρ*R*_11_^3^ vs the ring fraction *x*.

As for all mixtures considered, we have chosen *ab* > 1, phase separation always results. The overall density at which phase separation first sets in correlates with the magnitude of this product: indeed, the larger *ab* is, the earlier (in density) a transition takes place. It can further be seen that as the intermonomer attractions in the rings become stronger (λ_22_ growing as we move from A → B → D), the mixtures become more susceptible to phase separation, the effect being further enhanced by the increase of bending rigidity (βκ_2_ growing as we move from A → B → C → D). The tielines are almost horizontal, signaling that the phase transition is predominantly a demixing separation between two phases that are, roughly, equally dense.

#### 2.3.2 Linear-Catenane Mixtures

**Figure 5:**
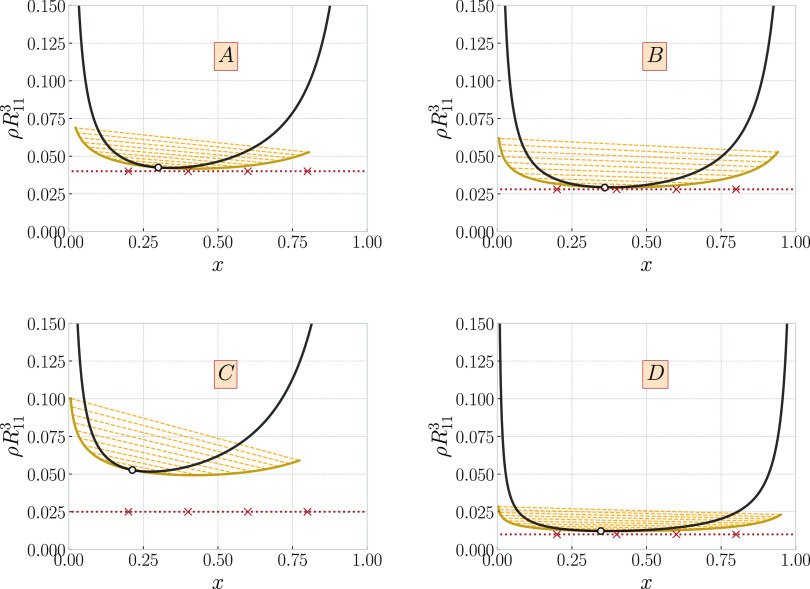
Phase diagrams for mixtures of long chains (*M*_1_ = 10) and polycatenanes (*M*_2_ = 20), plotted on the plane spanned by the total blob density and the fraction of rings, (ρ, *x*). The labels *A*–*D* on the panels correspond to the four selected cases from Table 1. Solid golden lines represent binodals, and solid black lines represent spinodals with critical points highlighted by a black point with white interior. Dashed golden lines correspond to selected tielines, connecting coexisting points on the binodals. Selected points marked by dark red crosses are further explored in Figure 7.

Here, we focus on binary mixtures of polycatenanes and long linear polymer chains. Specifically, we set the length of polycatenane chain to *M*_2_ = 20, while the linear polymer chain is comprised of 10 blobs: *M*_1_ = 10. In terms of the interaction potentials between the blobs, we consider the same four mixtures A, B, C, and D, as in the single blob case discussed earlier. The resulting phase diagrams are plotted in Figure 5, in the same variables and with the same symbols as in Figure 4 for the case where *M*_1_ = *M*_2_ = 1. It can be seen that the same trends also persist for the linear chain-polycatenane mixtures with the additional feature that now phase separation sets in at much lower overall densities of the blobs than in the previous case: polycatenane connectivity and longer chains enhance the phase separation propensity of the mixture, which retains its character as a demixing transition: indeed, the tielines remain also in this case roughly horizontal. Accordingly, tuning the effective potential between individual rings and chains and being able to control its characteristics via solvent quality and rigidity give us flexibility in steering the macrophase behavior of mixtures of polycatenanes and linear polymers.

### 2.4 Correlations and Response Functions

The minimization of the grand potential with respect to ρ_1_(**r**) and ρ_2_(**r**) yields equilibrium inhomogeneous density profiles for the two components. In what follows, we apply the above general DFT methodology to compute the bulk structural properties of ring–chain binary mixtures and the interfacial properties of these mixtures either in the bulk or in contact with a planar wall.

To characterize the structural properties of the binary mixture of rings and linear chains (*M*_1_ = *M*_2_ = 1), we first compute the inhomogeneous (spherically symmetric) density distributions ρ_*ij*_(*r*) of species *j* around a probe particle of species *i* fixed at the origin, which acts as an external potential *V*_*ij*_(*r*) on species *j*. The minimization of the grand potential βΩ of eq 7 gives the following result
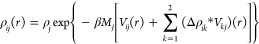
25where Δρ_*ik*_(*r*′) = ρ_*ik*_(*r*′) – ρ_*ik*_(*r*′ → ∞) and the star denotes the three-dimensional convolution
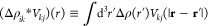
26Equation 25 is solved iteratively on a uniform grid with the spacing Δ*r* = 0.02*R*_11_, with numerical integration performed using 2-point Gaussian quadrature and employing a simple Picard iterative procedure. The boundary condition for the value of the density profile of species *j*, ρ_*ij*_(*r*), far away from the source particle of type *i* is set to the corresponding bulk partial density ρ_*j*_.

Using the standard Percus identity ρ_*ij*_(*r*) = ρ_*j*_g_*ij*_(*r*), we obtain the radial distribution functions *g*_*ij*_(*r*) between blobs *i* and *j*, as well as the corresponding total pair correlation functions *h*_*ij*_(*r*) = *g*_*ij*_(*r*)–1. We consider the Fourier space representation of the one-particle density of species (blob) ν, ρ̂^(ν)^(**k**), given by
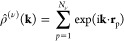
27where *N*_ν_ is the number of particles (blobs) of species ν and **r**_p_ is the position of particle p of that species. The response functions , *i*, *j* = 1, 2 are defined as
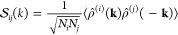
28and are related to the aforementioned pair correlation functions *h*_*ij*_(*r*) via
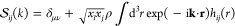
29where *x*_*i*,*j*_ = *N*_*i*,*j*_/*N* is the number fraction of species *i*, *j*.

In the vicinity of phase transitions for binary mixtures, it is relevant to explore which is the fluctuating quantity that reacts more sensitively to the incipient instability. In particular, the issue is to distinguish between particle number fluctuations and composition fluctuations, which are embodied in the long-wavelength limit of the number–number and concentration–concentration response functions  and , which are defined by eqs 30 and 31, respectively
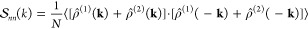
30and
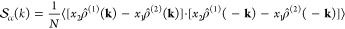
31These response functions are related to the *S*_*ij*_(*k*) via^112,113^
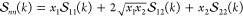
32and
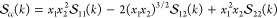
33We emphasize that here we call “species” the linear or ring blobs. For linear-ring mixtures (*M*_1_ = *M*_2_ = 1), they do indeed coincide with the individual polymers in solutions, but for linear-catenane mixtures (*M*_1_ > 1, *M*_2_ > 1), they do not. It is for this purpose that we call the quantities  with the generic name “response functions” to take both cases into consideration, since they are indeed expressing the response of some quantity in the system to an external potential caused by a blob at the origin. For *M*_1_ = *M*_2_ = 1, they coincide with the structure factors^84,112,113^*S*_μν_(*k*) and we use the name and symbol for this particular case, whereas we employ the symbol *Q*_μν_(*k*) for the case *M*_1_ > 1, *M*_2_ > 1 in what follows.

**Figure 6:**
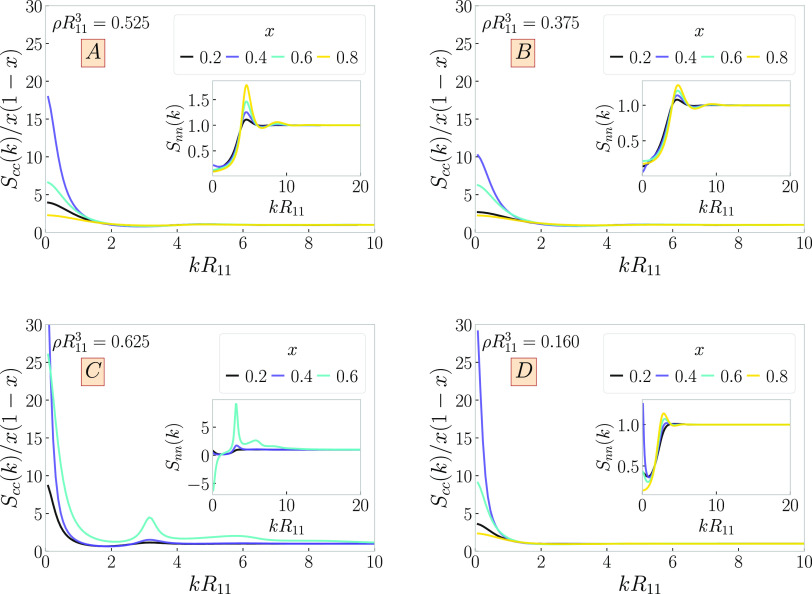
Concentration–concentration structure factors *S_cc_*(*k*) from eq 31 (main plots) and the number–number structure factors *S_nn_*(*k*) from eq 30 (insets) for mixtures of linear chains (*M*_1_ = 1) and rings (*M*_2_ = 1). The labels (*A–D*) on the panels correspond to the four selected cases from Table 1. We show the functions for various ring fractions at selected densities and ring fractions, corresponding to the dark red crosses in Figure 4.

We have evaluated the above-mentioned correlation functions in *r*-space and the concomitant response functions in *k*-space for all points marked with red crosses in Figures 4 and 5. For ring–chain binary mixtures, the concentration–concentration structure factors *S_cc_*(*k*) for the binary mixtures A, B, C, and D are presented in Figure 6, with insets showing the results for the number–number *S_nn_*(*k*). In all cases, it is the former quantity that shows a strong response at the long-wavelength limit (*k* → 0), whereas the latter remains small. This is a clear signal that the phase transition is of the demixing type since high values of *S_cc_*(*k* → 0) point to strongly enhanced long-wavelength concentration fluctuations, whereas the corresponding number fluctuations remain suppressed. An exception is the second point of system D, marked *x* = 0.4, which lies very close to the critical point of the system, on which all response functions diverge.

**Figure 7:**
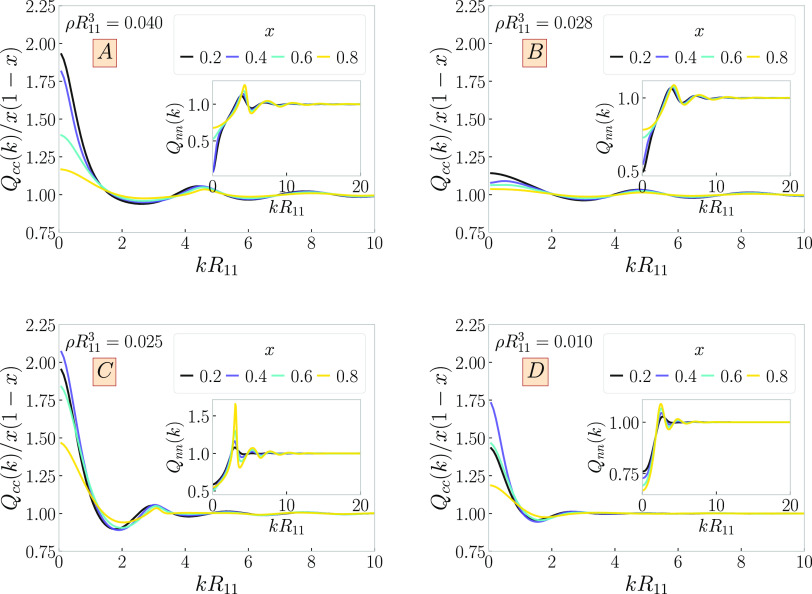
Concentration–concentration response functions *Q_cc_*(*k*) and their number–number counterparts *Q_nn_*(*k*) from eq 30 (insets), for mixtures of long linear chains (*M*_1_ = 10) and polycatenanes (*M*_2_ = 20). The labels (*A–D*) on the panels correspond to the four selected cases from Table 1. We show the functions for various ring fractions at selected densities and ring fractions, corresponding to the dark red crosses in Figure 5.

Similar results are obtained for the response functions *Q_cc_*(*k*) and *Q_nn_*(*k*) of the chain-catenane mixtures shown in Figure 7. As the points considered on the phase diagram are sufficiently far removed from the actual critical points of the single blob mixtures, the low *k* peaks in *S_cc_*(*k*) are much less pronounced compared to the individual blob case shown in Figure 6. Moreover, here we also obtain a *Q_nn_*(*k*) response function for the point marked *x* = 0.8 of system C, which shows a strong propensity of the system to modulate with a finite wavenumber k_*_. This is the case of a nearby-lying λ-line of the catenane, which signals the tendency of the system to order into a crystal, and which interferes with the spinodal line of the macrophase separation, as also found in previous work.^105^

### 2.5 Free Interface

**Figure 8:**
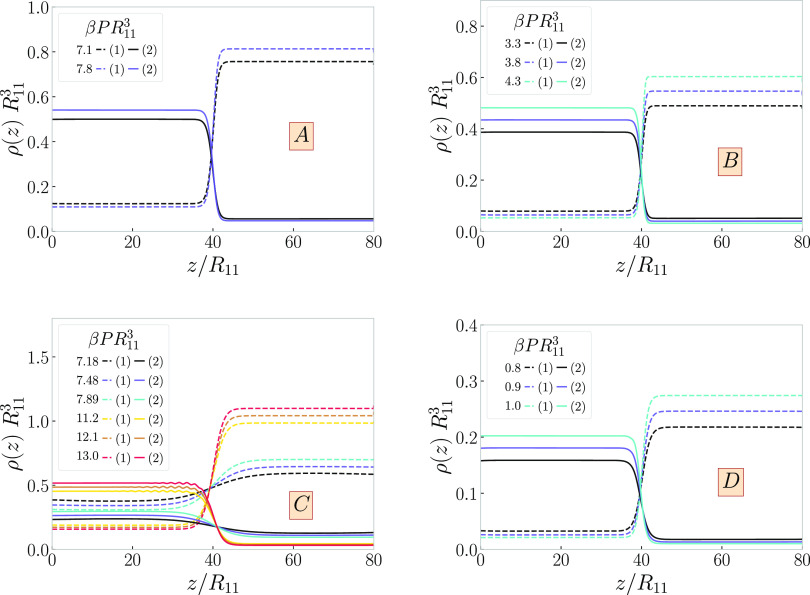
Interface density profiles for solutions of linear chains (*M*_1_ = 1) and rings (*M*_2_ = 1) as a function of the position *z* vertical to the interface. The labels (*A–D*) on the panels correspond to the four selected cases from Table 1. We show the profiles for different values of the pressure, as indicated in the legends, corresponding to the dark red tielines from Figure 4. Dashed curves denote ρ_1_(*z*) and solid ones ρ_2_(*z*). The boundary conditions are chosen in such a way that we have the coexisting phase of the left end point of the tieline at *z* → +∞ and that of the right end point at *z* → −∞.

In this section, we restrict ourselves to linear chain/ring mixtures, i.e., *M*_1_ = *M*_2_ = 1. The presence of two coexisting phases on the two sides of the binodals implies that a free interface between the two will form when the two chemical potentials attain the appropriate values. Let therefore I and II denote the two coexisting phases with partial densities (ρ_1_^I^, ρ_2_^I^) and (ρ_1_^II^, ρ_2_^II^), respectively. The density profiles resulting into a free interface can be calculated by minimizing the grand potential, eq 7 under the boundary conditions
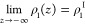
34
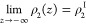
35
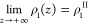
36
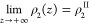
37i.e., forcing the bulk phase I at *z* → – ∞ and bulk phase II at *z* → +∞. Defining μ̅_*i*_ ≡ μ_*i*_ – 3 ln(Λ_*i*_/*R*_11_), the self-consistent equations for the density profiles ρ_*i*_(*z*) read as
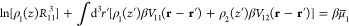
38
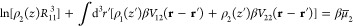
39To determine the free interfaces, eqs 38 and 39 are solved iteratively, starting from a smooth function (e.g., a hyperbolic tangent form) fulfilling the boundary conditions for any chosen binodal and iterated until convergence is achieved. Results are shown in Figure 8 for selected binodals in the systems A–D. In all cases, the profiles are monotonic, i.e., devoid of the strong correlation peaks usual for steeply diverging interaction potentials, since the effective potentials at hand are soft and penetrable. An exception to this is the ring profiles for the higher pressure values in panel C. There, the ring-rich phase displays weak oscillations indicative of the nearby-lying λ̇-line of species 2, as mentioned also above for the response functions of the same.

As, evidently, the density profiles only depend on the direction *z* perpendicular to the planar, free interface, the grand potential in this case takes the form Ω_*lv*_ = *L*^2^ ∫ d*z* ω_*lv*_(*z*), with the area *L*^2^ of the system perpendicular to the interface and the liquid–vapor grand potential density ω_*lv*_(*z*). The liquid–vapor interfacial tension γ_*lv*_ results as the integrated difference between ω_*lv*_(*z*) and the bulk grand potential density  as
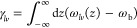
40

### 2.6 Wetting of a Planar Wall

As a second application of the inhomogeneous DFT formalism, we will study the wetting properties of ring–chain binary mixtures (*M*_1_ = *M*_2_ = 1) at a structureless planar wall located in the *xy* plane. To model the wall–fluid potentials, we employ the same functional forms as in earlier study of wetting behavior of soft binary mixtures^94^
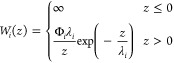
41where *i* = {1, 2} and we set βΦ_1_ = 1, λ_1_ = *R*_11_, and λ_2_ = *R*_12_, while βΦ_2_ (the dimensionless strength of the ring–wall interaction) is varied in order to control the wetting behavior of the mixture at the wall. As the wall–fluid potentials are only functions of distance *z* from the wall, the same applies to the equilibrium density profiles of ρ_*i*_(*z*) of both components. By minimizing βΩ and replacing the chemical potentials μ_*i*_ in favor of the bulk densities ρ_*i*_ = ρ_*i*_(*z* = ∞), one obtains the equilibrium density profiles in inhomogeneous binary mixtures^94^
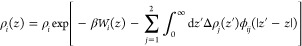
42where Δ*ρ*_*i*_(*z*) = ρ_*i*_(z) – ρ_*i*_(*z* = ∞) and
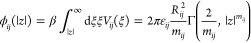
43with Γ(*a*, *x*) being the incomplete γ function. Then, eq 42 is solved iteratively on a uniform grid with the spacing Δ*z* = 0.02*R*_11_, with numerical integration performed using 2-point Gaussian quadrature and employing a simple Picard iterative procedure.

For studying the wetting behavior, the DFT calculations are performed at coexistence, where one of the two coexisting phases (which we arbitrarily call “liquid” phase) is rich in the linear polymers and poor in ring ones, while the other (“vapor”phase) is rich in the ring polymers and poor in linear ones. Accordingly, by scanning the parameter βΦ_2_ from low to high values, i.e., making the wall–ring potential progressively more repulsive, one can go from “drying” to “wetting” as the “liquid” (ring-poor phase) intervenes between wall and vapor to keep the ring-poor phase closer to the wall and the ring-rich “vapor” phase away from it.

The key observable for studying the wetting behavior is the contact angle θ given by Young’s equation^114^
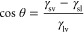
44where γ_sv_, γ_sl_, and γ_lv_ are the interfacial tensions between the solid and the “vapor” phase, between the solid and the “liquid” phase, and between “liquid” and “vapor” phases. In order to calculate the two solid–fluid tensions, one sets the boundary condition far away from the wall to the corresponding bulk densities and computes the inhomogeneous density profiles ρ_*i*_(*z*) from DFT as described above. From these density profiles and eq 7, one can readily compute the grand potential density βω(*z*), which yields the solid–fluid interfacial tension
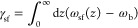
45where *f* = *l*, *v* is the subscript and ω_b_ is the bulk value of the grand potential density. The analogous expression holding for the liquid–vapor interfacial tension γ_lv_ is given in eq 40.

**Figure 9:**
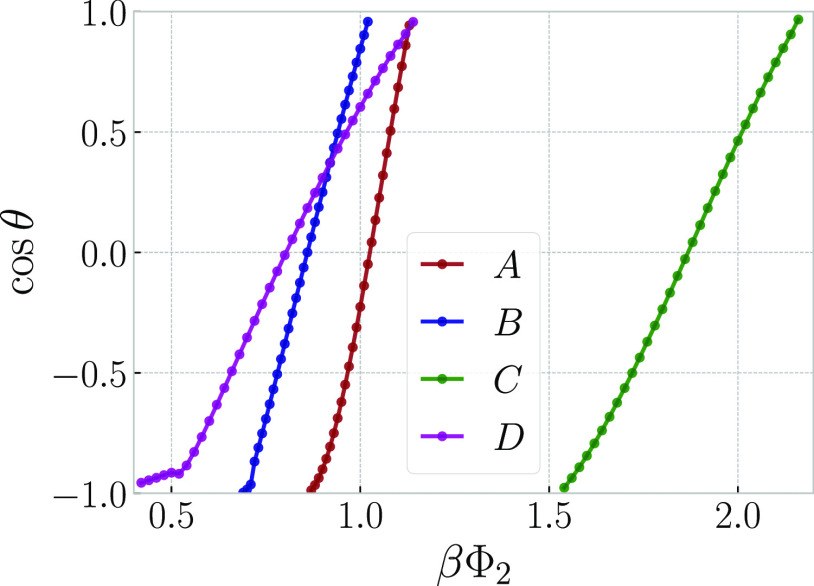
Cosine of the contact angle between a planar wall and a liquid drop (linear-chain-rich phase) in coexistence with the vapor (ring-polymer-rich phase), as a function of the strength βΦ_2_ of the wall–ring interaction for systems *A*, *B*, *C*, and *D* as indicated in the legend. The corresponding pressures are *A*: 2*P*_c_; *B*: 2*P*_c_; *C*: 1.4*P*_c_; and *D*: 2*P*_c_, where *P*_c_ denotes the pressure at the critical point.

For studying the wetting behavior at a planar wall, DFT calculations are performed for a single chosen pressure for each of the four binary mixtures. Specifically, we have selected *P* = 2*P*_c_ for the systems *A*, *B*, and *D*, and *P* = 1.4*P*_c_ for the system *C*. For these pressures, we compute the liquid–vapor interfacial tension γ_lv_ from eq 40 and the solid–liquid interfacial tension γ_sl_ from eq 45, with analogous equation used to compute the solid–vapor interfacial tension γ_sv_. The latter two calculations are performed for a range of wall–ring interaction strength βΦ_2_. Having obtained the three interfacial tensions, we computed the contact angle from eq 44; the corresponding results are shown in Figure 9 as a function of βΦ_2_. One sees that for all four systems, the corresponding lines cross the line cos θ = 1 at a finite angle, indicating the occurrence of a first-order wetting transition.^100^

## 3 Conclusions

We have applied coarse-graining techniques and density functional theory to examine the phase behavior and bulk and interfacial structures of binary mixtures between linear chains and ring polymers as well as of mixtures of linear chains and polycatenanes. For the latter case, the polycatenane has been described once again in a coarse-grained fashion as a succession of ring blobs, without further specification of the links between the same; in other words, we have ignored any difference between rings connected to one another by mechanical Hopf links (i.e., true polycatenanes) and successions of rings bonded with one another via chemical bonds. Recent work has shown that there are differences between the two regarding the values of the resulting single-molecule elastic modulus^39^ but we do not anticipate any serious effect of the latter on the phase behavior and in particular on the demixing propensity of polycatenanes and linear chains.

As a general trend, a worsening solvent quality for the ring blobs leads to a tendency for a demixing transition between the linear and the ring components, showing the same trend for the corresponding catenanes of rings with chains. On the other hand, increasing the stiffness of the ring induces no clear, monotonic trend on the demixing propensity, as the net effect depends on a combination of solvent quality and ring stiffness in a nontrivial way. Nevertheless, our theory allows for a clear prediction of the phase behavior on the basis of the three effective interactions *V*_*ij*_(*r*), which lead to the determination of the quantities *a* and *b* of eq 18: if the product *ab* > 1, demixing will occur, but if *ab* ≤ 1, the system will remain mixed at all densities.

Although the topology of the rings does enter the formalism indirectly through the shape and form of the resulting effective potentials, at the blob picture, certain important details of the spatial organization of the system drop out of sight. Such features are, e.g., the frequency and depth of threadings between chains and rings, the knotting of the linear chain or the catenanes, and the effects that these have on the dynamics and the relaxation of the mixture as well as on the demixing dynamics of the latter. To illuminate such issues, detailed computer simulations and experimental investigations are necessary.

## Supporting Information Available

Effective potentials for different setups of parameters and plot showing positions of critical points for catenanes of different lengths and table listing the parameters of the studied systems (PDF)
