# Supplementary material for: Mixing Linear Polymers with Rings and Catenanes: Bulk and Interfacial Behavior
Source: Macromolecules. 2023 Oct 3;56(20):8168–82. doi: 10.1021/acs.macromol.3c01267 (PMC10601540; doi:10.1021/acs.macromol.3c01267)
Supplement: Supplementary file 1 — ma3c01267_si_001.pdf [file ma3c01267_si_001.pdf]

Supporting Information: Mixing Linear Polymers with Rings and Catenanes: Bulk and Interfacial Behavior

Roman Staňo,[*,†,‡] Christos N. Likos,[*,†] and Sergei A. Egorov[*,¶,§]

†*Faculty of Physics, University of Vienna, Boltzmanngasse 5, 1090 Vienna, Austria*

‡*Vienna Doctoral School in Physics, University of Vienna, Boltzmanngasse 5, 1090 Vienna, Austria*

¶*Department of Chemistry, University of Virginia, Charlottesville, Virginia 22901, USA*

§*Erwin Schrödinger International Institute for Mathematics and Physics, Boltzmanngasse 9, 1090 Vienna, Austria*

E-mail: roman.stano@univie.ac.at; christos.likos@univie.ac.at; sae6z@virginia.edu

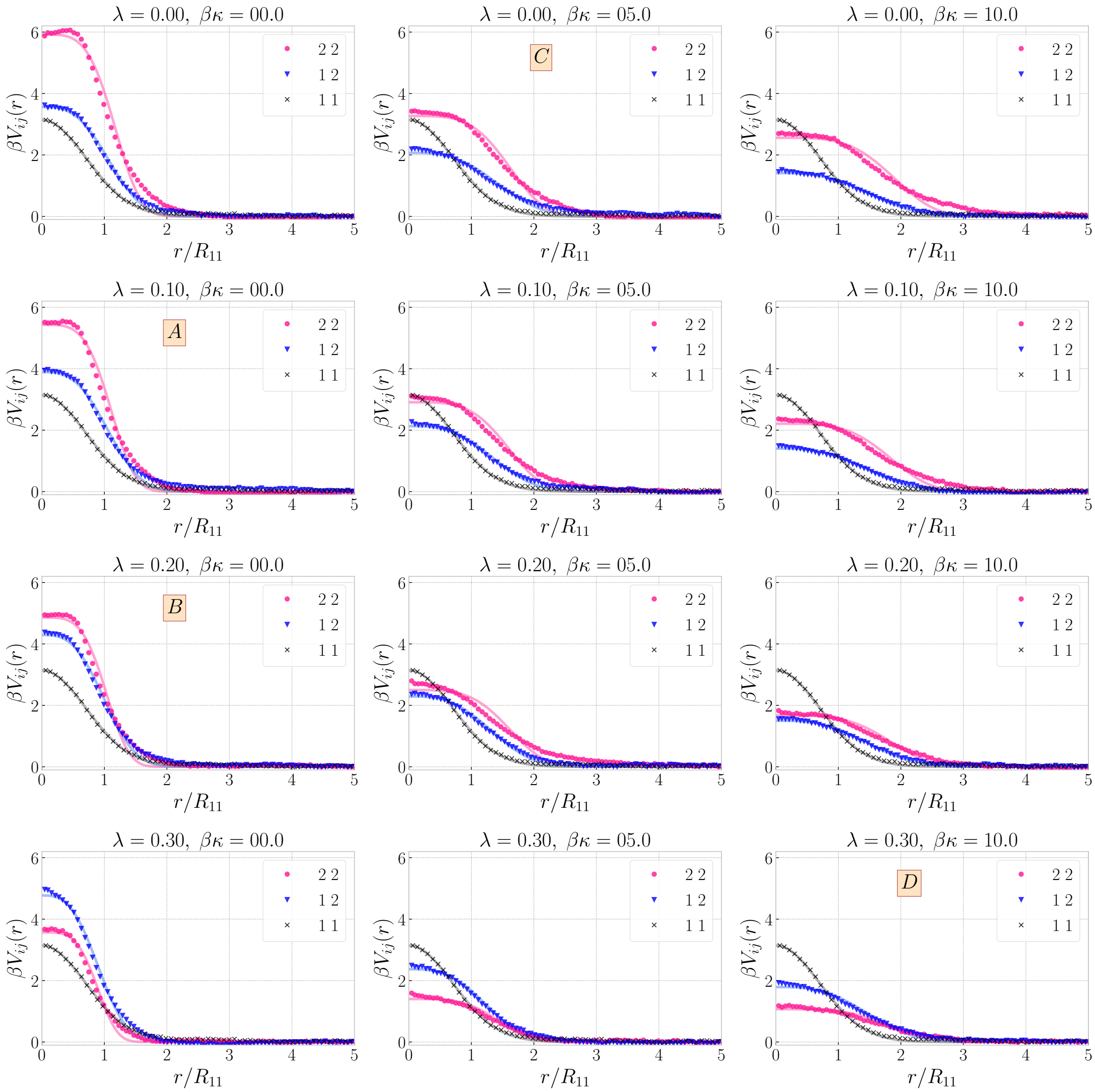

Figure S1: Effective isotropic potentials between two rings (22), two chains (11) and ring and chain (12) as a function of separation between the two molecules, plotted for all the explored cases from Tab. S1. Labels $A - D$ denote the cases from Tab. 1 in the main text. Points were obtained by monomer-resolved simulations and lines are fits. Distance is normalized by the characteristic length scale of chain-chain interaction, R_{11}.

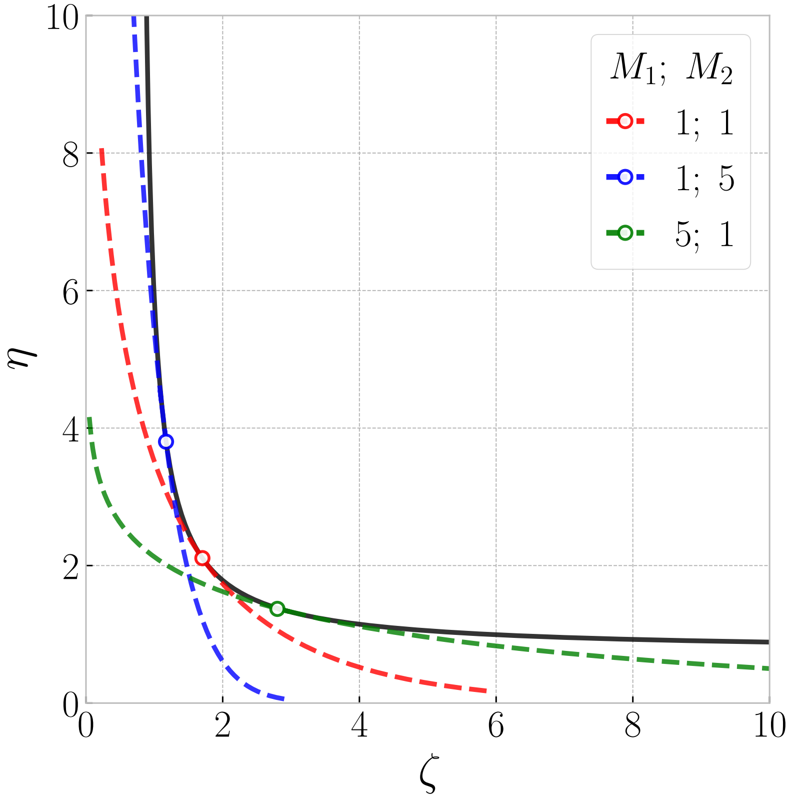

Figure S2: Phase diagrams for the system D plotted in the plane of two dimensionless variables $\zeta = M_1\rho_1\beta\hat{V}_{11}(0)$ and $\eta = M_2\rho_2\beta\hat{V}_{22}(0)$. Dashed lines are binodals plotted for different combinations of (M_1, M_2) with their respective critical point marked by the point with the white interior. All the critical points belong to the universal spinodal plotted in black.

Table S1: Table listing the parameters of the studied systems: stiffness of chain (1), $\beta\kappa_1$, and ring (2), $\beta\kappa_2$, depth of the interaction well between monomeric U_{ij} for chain-chain, λ_{11}, chain-ring, λ_{12}, and ring-ring, λ_{22}, followed by the fitted parameters of the resultant effective potential and mixing criterion ab.

	$U_{\text{bend}}(\phi)$		$U_{ij}(s)$			$V_{11}(r)$		$V_{12}(r)$		$V_{22}(r)$		
Case	$\beta\kappa_1$	$\beta\kappa_2$	λ_{11}	λ_{12}	λ_{22}	$\beta\varepsilon_{11}$	R_{11}/σ	$\beta\varepsilon_{12}$	R_{12}/σ	$\beta\varepsilon_{22}$	R_{22}/σ	ab
	0	0	0.0	0.0	0.0	3.14	5.55	3.56	6.81	5.92	7.07	0.924
A	0	0	0.0	0.0	0.1	3.14	5.55	3.89	6.73	5.43	6.69	1.321
B	0	0	0.0	0.0	0.2	3.14	5.55	4.29	6.34	4.86	6.25	1.536
	0	0	0.0	0.0	0.3	3.14	5.55	4.77	5.74	3.57	5.40	2.203
C	0	5	0.0	0.0	0.0	3.14	5.55	2.07	9.07	3.27	9.74	1.212
	0	5	0.0	0.0	0.1	3.14	5.55	2.13	8.58	2.91	9.60	1.073
	0	5	0.0	0.0	0.2	3.14	5.55	2.29	8.39	2.50	9.73	1.210
	0	5	0.0	0.0	0.3	3.14	5.55	2.37	7.84	1.40	8.67	2.187
	0	10	0.0	0.0	0.0	3.14	5.55	1.41	9.67	2.56	11.42	0.651
	0	10	0.0	0.0	0.1	3.14	5.55	1.40	9.41	2.21	11.11	0.681
	0	10	0.0	0.0	0.2	3.14	5.55	1.50	9.41	1.67	11.10	1.040
D	0	10	0.0	0.0	0.3	3.14	5.55	1.79	9.40	1.07	11.03	2.340
